# Supplementary material for: The cytoplasmic phosphate level has a central regulatory role in the phosphate starvation response of Caulobacter crescentus
Source: Commun Biol. 2024 Jun 26;7:772. doi: 10.1038/s42003-024-06469-y (PMC11208175; doi:10.1038/s42003-024-06469-y)
Supplement: Supplementary file 2 — Supplementary Information [file 42003_2024_6469_MOESM2_ESM.pdf]

Supplementary information

The cytoplasmic phosphate level has a central regulatory role in the phosphate starvation response of *Caulobacter crescentus*

Maria Billini, Tamara Hoffmann, Juliane Kühn, Erhard Bremer, Martin Thanbichler

SUPPLEMENTARY FIGURES

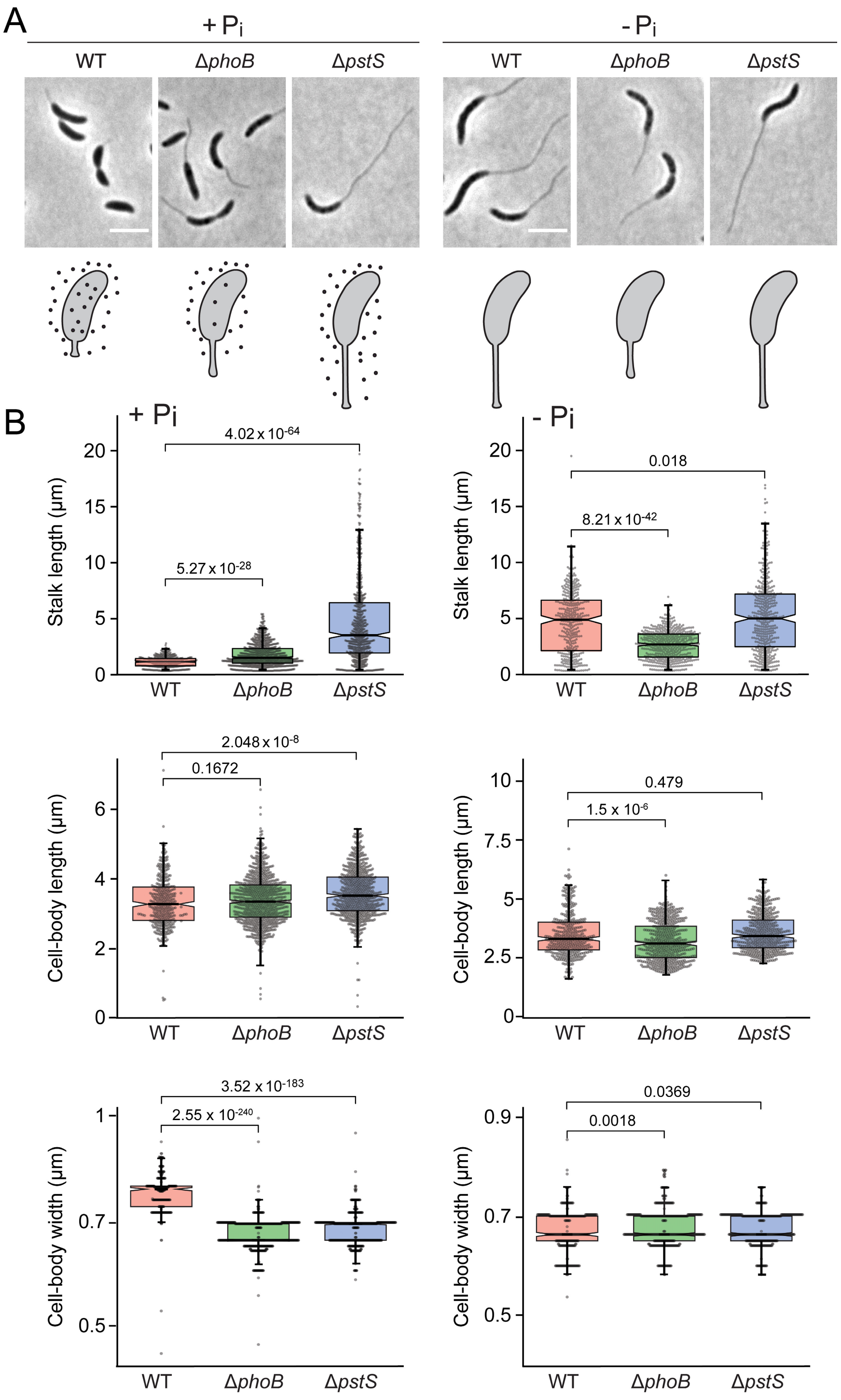

Supplementary Figure 1. Phenotypes of different *C. crescentus* strains under phosphate starvation. (A) Phase contrast images of *C. crescentus* wild-type (CB15N), Δ*phoB* (JK2) and Δ*pstS* (JK158) cells grown to exponential phase in PYE medium (+ P_i) or grown in PYE medium, diluted 1:20 in M2G^{-P} medium (- P_i) and then incubated for another 24 h prior to analysis. Scale bar: 3 µm. The schematics at the bottom illustrate the levels of phosphate (black dots) in cytoplasm of the respective strains. **(B)** Combined beeswarm and box plots representing the distribution of stalk lengths as well as cell-body lengths and widths in cultures of the strains shown in panel A. The boxes give the interquartile range, the notches indicate the median values, and the whiskers extend to the 5th and 95th percentile. Number of cells measured: WT (387), Δ*phoB* (841), Δ*pstS* (692) in PYE medium (+ P_i) and WT (373), Δ*phoB* (502), Δ*pstS* (446) in M2G^{-P} medium (- P_i). Numbers indicate the statistical significance (*p* values) of differences between strains (two-tailed, unpaired t-test).

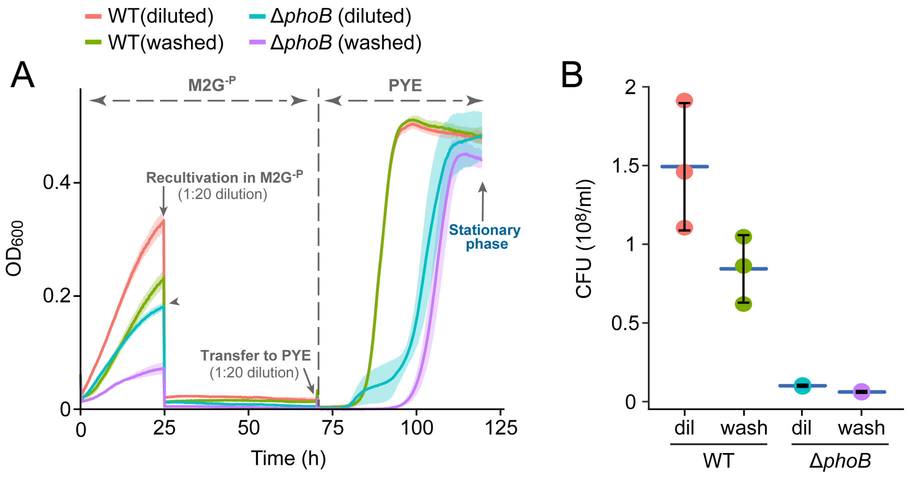

Supplementary Figure 2. Reduced growth and fitness of Δ*phoB* cells in phosphate-limited medium. (A) Growth curves of wild-type (CB15N) and Δ*phoB* (JK2) cells under different conditions. Cultures were grown to stationary phase in PYE medium (phosphate-rich) and either immediately diluted 1:20 into M2G^{-P} medium or first washed three times with M2G^{-P} medium before dilution. After 25 h, they were diluted (1:20) again into new M2G^{-P} medium and incubated for another 48 h. Subsequently, the cultures were diluted (1:20) in PYE medium and incubated until they reached stationary phase. Lines represent the mean of three independent experiments. Shades indicate the standard deviation. **(B)** Fitness of cells during phosphate starvation. Equal amounts of wild-type and Δ*phoB* mutant cells were either washed three times with M2G^{-P} medium or directly diluted (1:20) in M2G^{-P} medium and incubated for 72 h. Afterwards, cells were diluted and plated on PYE agar plates to determine the number of colony-forming units. Colonies were counted after two (WT) or four (Δ*phoB*) days. Data represent the number of colony-forming units (CFU) obtained in three independent experiments.

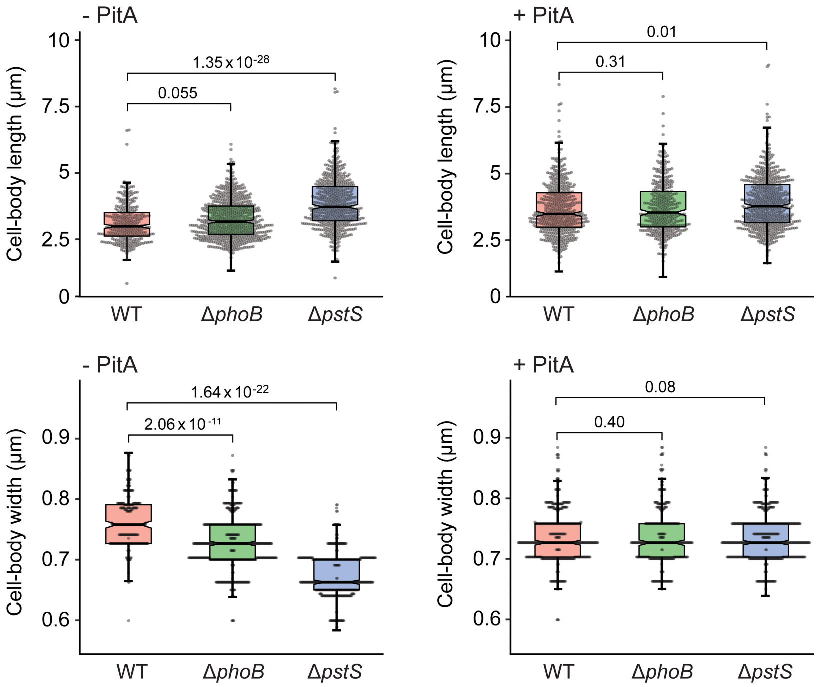

Supplementary Figure 3. Cell-body morphology of different *C. crescentus* strains in the absence or presence of PitA. Shown are combined beeswarm and box plots representing the distribution of cell-body lengths and widths in cultures of the strains shown in **Figure 4A.** The boxes give the interquartile range, the notches indicate the median values, and the whiskers extend to the 5th and 95th percentile. Number of cells measured: WT (313), Δ*phoB* (622), Δ*pstS* (519) in the absence of xylose (- PitA) and WT (485), Δ*phoB* (372), Δ*pstS* (567) in the presence of xylose (+ PitA). Numbers indicate the statistical significance (*p* values) of differences between strains (unpaired, two-tailed t-test).

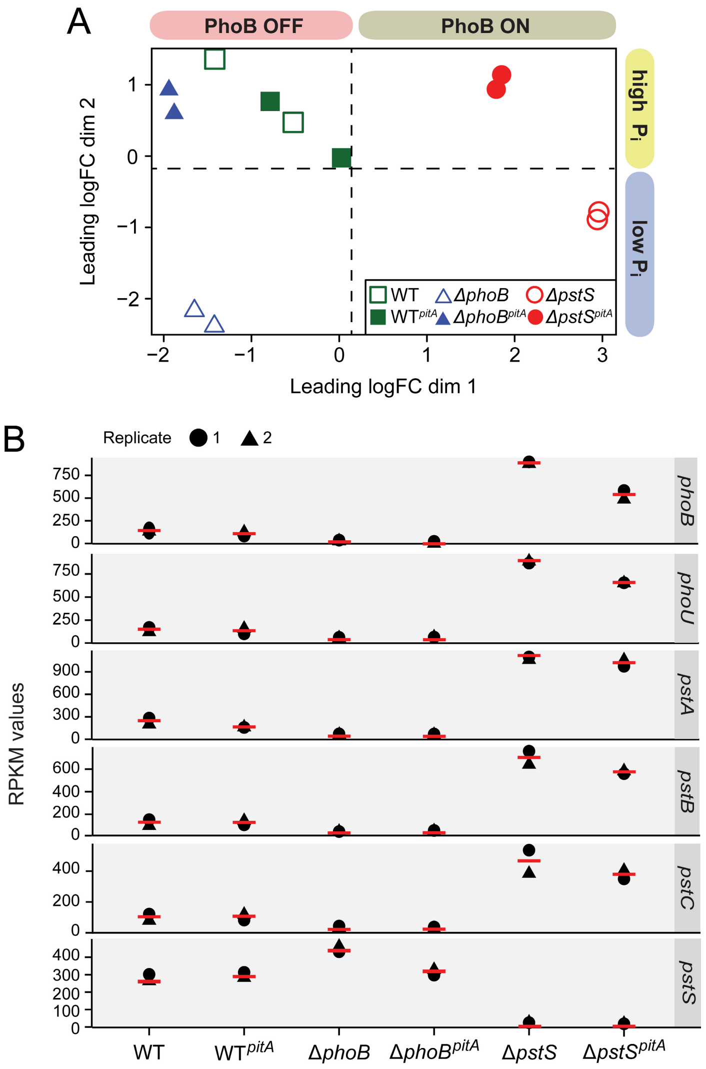

Supplementary Figure 4. Control of the quality of the transcriptome data. (A) Multi-dimensional scaling (MDS) plot showing the degree of similarity between the RNA-seq datasets obtained for the WT (CB15N), WT^{pitA} (MAB257), Δ*phoB* (JK2), Δ*phoB*^{pitA} (MAB258), Δ*pstS* (JK158) and Δ*pstS*^{pitA} (MAB259) strain. Each strain was analyzed in duplicate. logFC: $\log_2$ of the fold change. **(B)** Plots showing the RPKM values obtained for the *pstC*, *pstA*, *pstB*, *phoU*, *phoB* and *pstS* genes in the indicated strains. Each strain was analyzed in duplicate. Red lines indicate the average RPKM value in each condition.

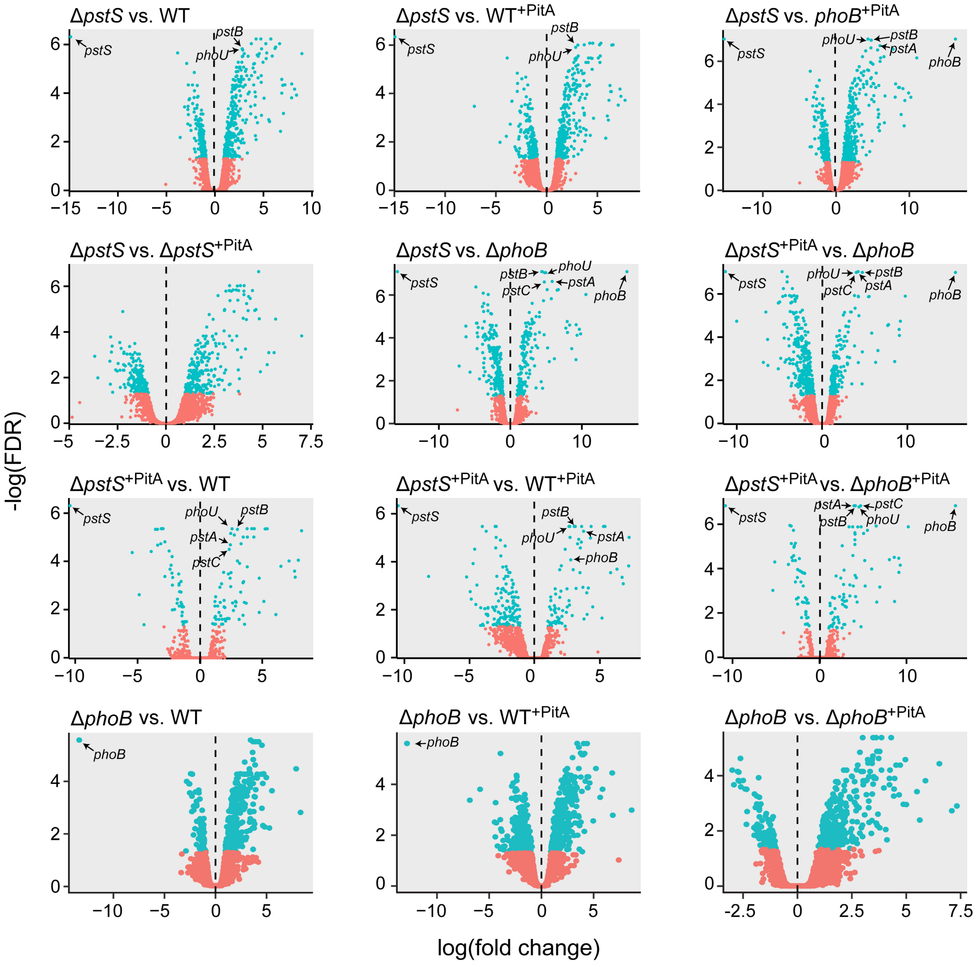

Supplementary Figure 5. Volcano plots showing the pairwise comparisons between RNA-seq datasets used in this study. For each open reading frame, the negative $\log_{10}$ value of the false-discovery rate (FDR) was plotted against the $\log_2$ of the fold change of the respective transcript. Orange and cyan color indicates an FDR that lies below or above a significance threshold of 0.05, respectively. The data points corresponding to the *pstC*, *pstA*, *pstB*, *phoU*, *phoB* and *pstS* genes are highlighted to facilitate the comparison of the different datasets.

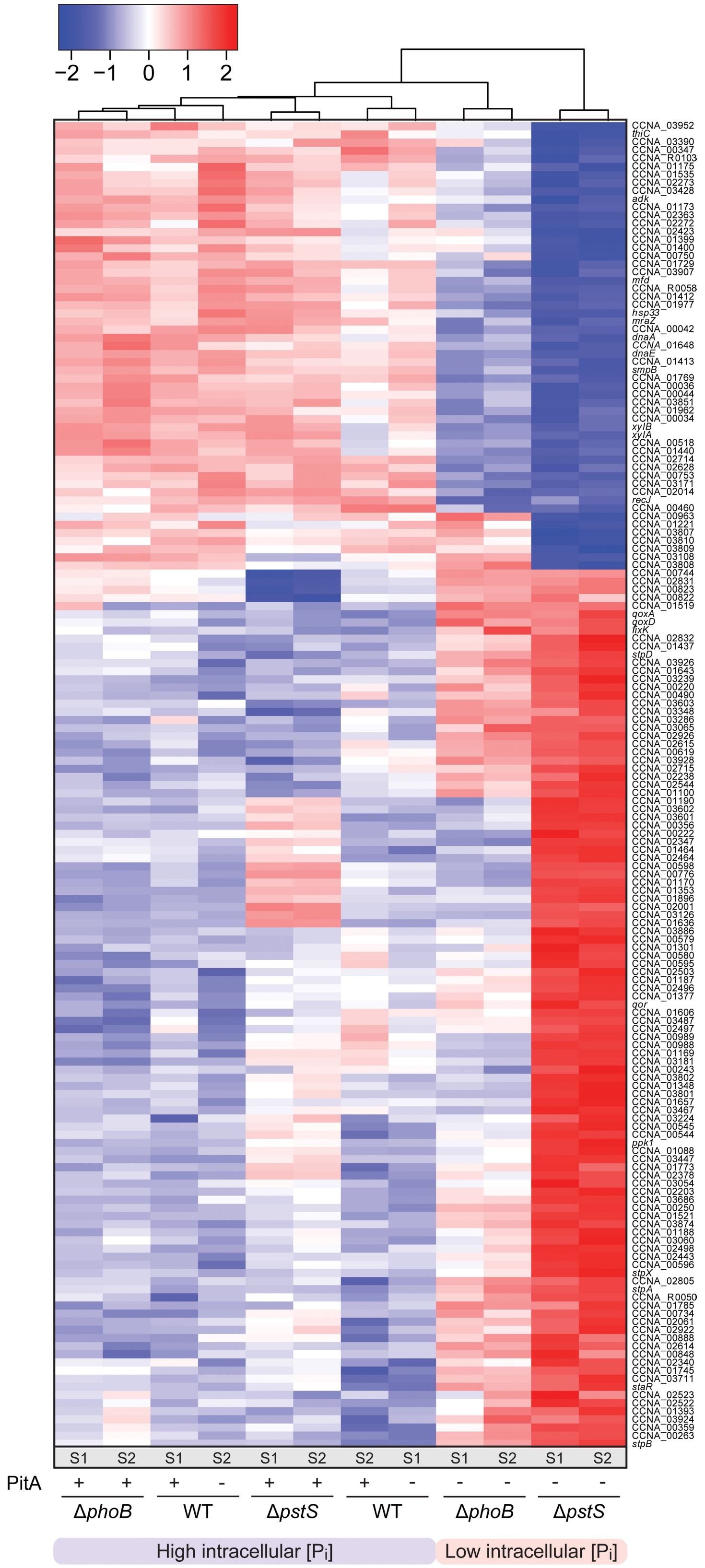

Supplementary Figure 6. Clustering analysis comparing the expression levels of the 163 genes that respond only to a severe decrease in the cytoplasmic phosphate level (in the presence of PhoB) in different strain backgrounds. White color represents the average transcript level of each gene among the tested condition. Red and blue color indicates an increase or decrease, respectively, in the transcript levels compared to the average. Normalized logCPM values were used in each case, leading to a fixed range of values for all genes. S1 and S2 indicate the two replicates analyzed for each strain.

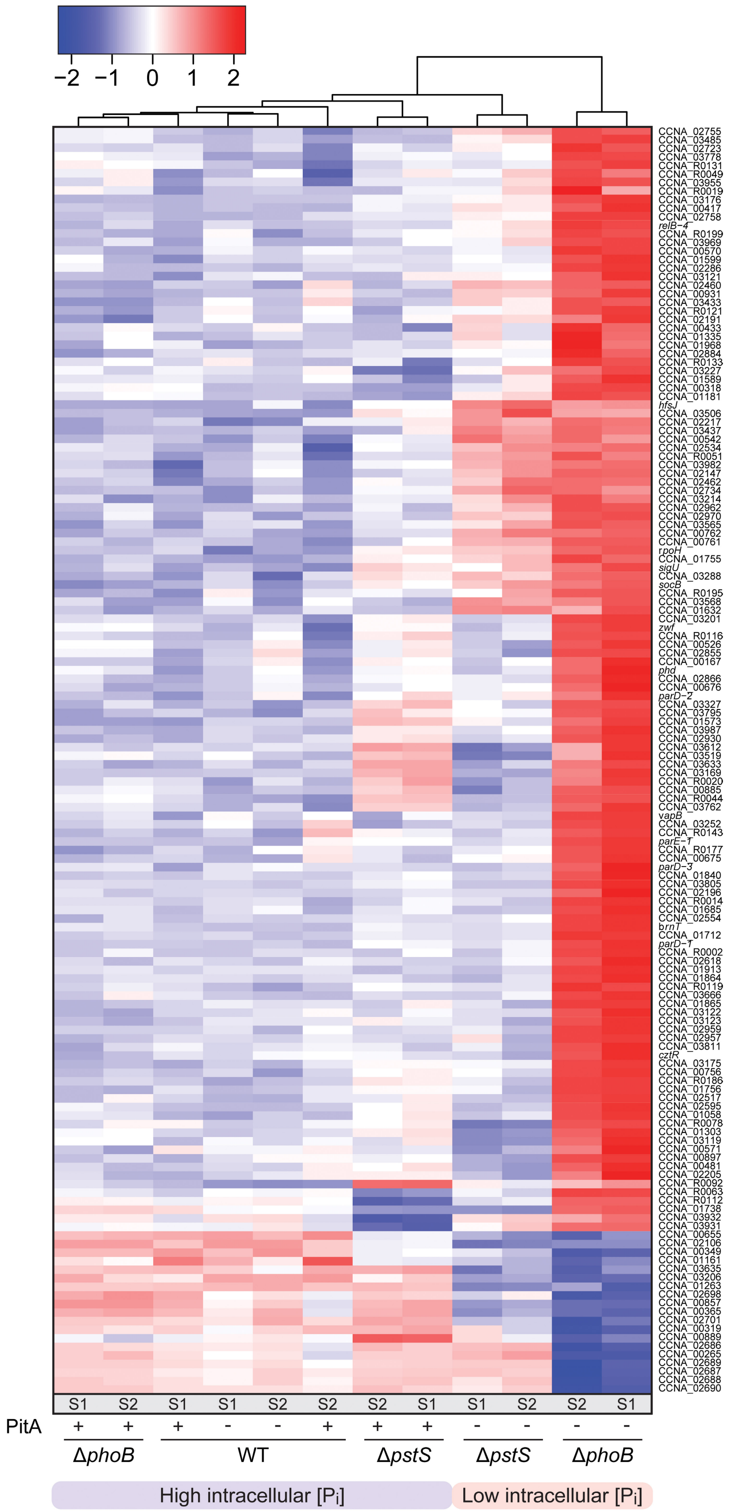

Supplementary Figure 7. Clustering analysis comparing the expression levels of the 148 genes that respond only to moderate changes in the cytoplasmic phosphate level (in the absence of PhoB) in different strain backgrounds. White color represents the average transcript level of each gene among the tested condition. Red and blue color indicates an increase or decrease, respectively, in the transcript levels compared to the average. Normalized logCPM values were used in each case, leading to a fixed range of values for all genes. S1 and S2 indicate the two replicates analyzed for each strain.

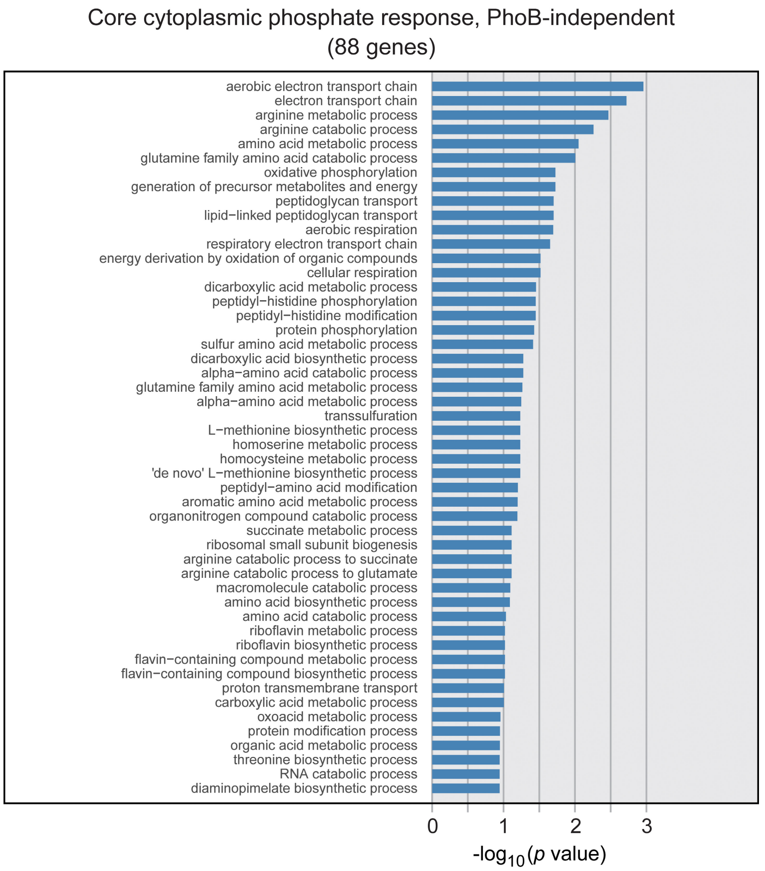

Supplementary Figure 8. Bar charts representing the enrichment of top-scoring GO ontology terms among the 88 genes that respond robustly to changes in the cytoplasmic phosphate level in a manner independent of PhoB (see **Figure 7C).** The bars represent the negative $\log_{10}$ of the p values (Fisher test) determined for each GO term. A list of all GO terms is provided in **Supplementary Data 3**.

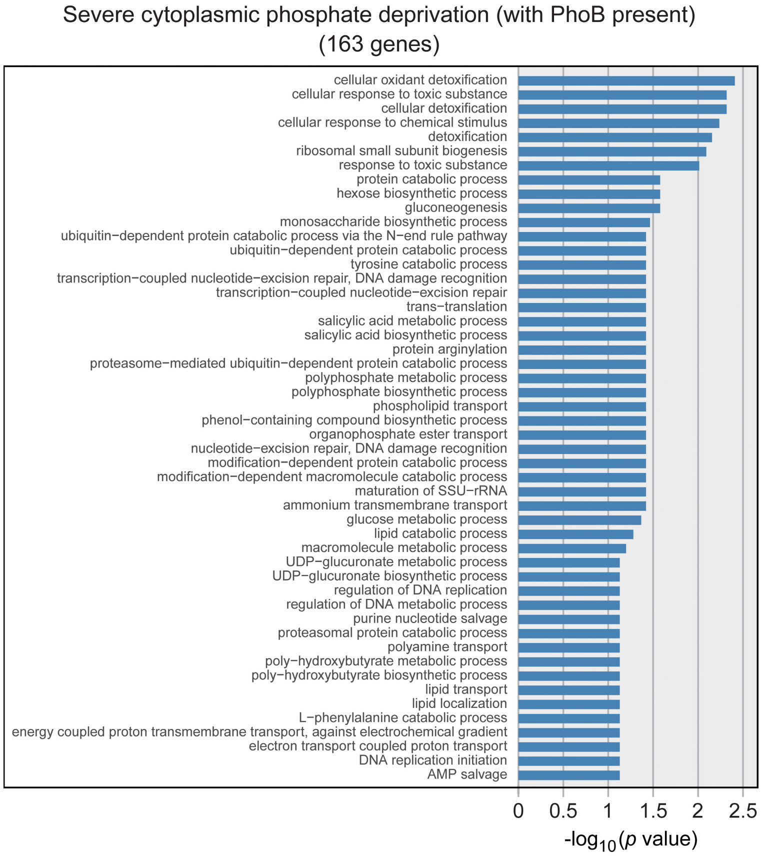

Supplementary Figure 9. Bar charts representing the enrichment of top-scoring GO ontology terms among the 163 genes that respond only to severe cytoplasmic phosphate depletion (with PhoB present) (see **Figure 7A**). The bars represent the negative $\log_{10}$ of the *p* values (Fisher test) determined for each GO term. A list of all GO terms is provided in **Supplementary Data 3**.

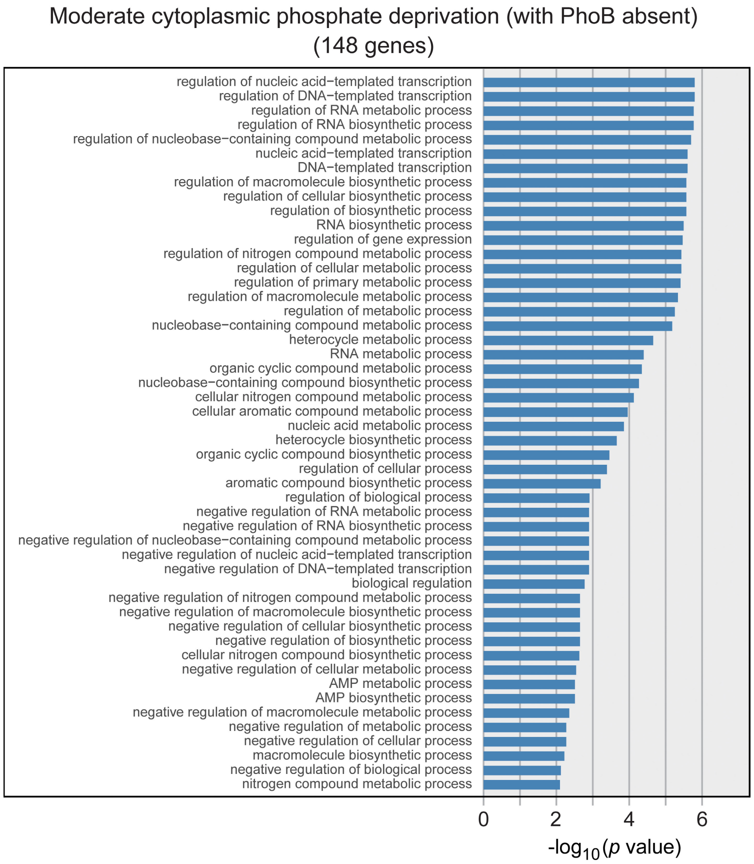

Supplementary Figure 10. Bar charts representing the enrichment of top-scoring GO ontology terms among the 148 genes that respond only to moderate cytoplasmic phosphate depletion (with PhoB absent) (see **Figure 7B**). The bars represent the negative $\log_{10}$ of the *p* values (Fisher test) determined for each GO term. A list of all GO terms is provided in **Supplementary Data 3**.

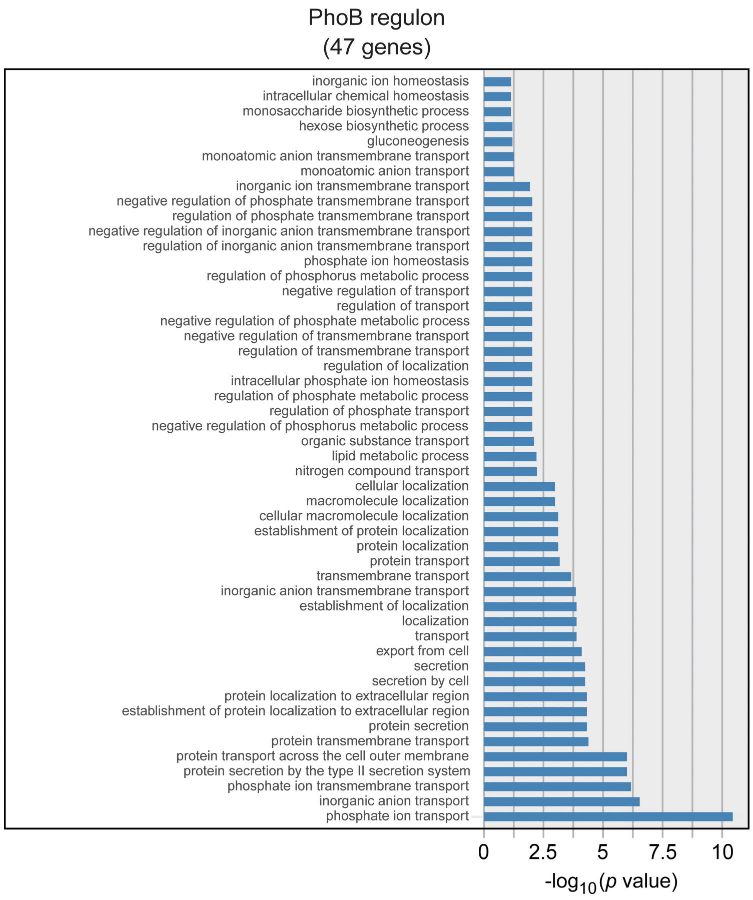

Supplementary Figure 11. Bar charts representing the enrichment of top-scoring GO ontology terms among the 47 genes of the PhoB regulon (see **Figure 8C**). The bars represent the negative $\log_{10}$ of the p values (Fisher test) determined for each GO term. A list of all GO terms is provided in **Supplementary Data 3**.

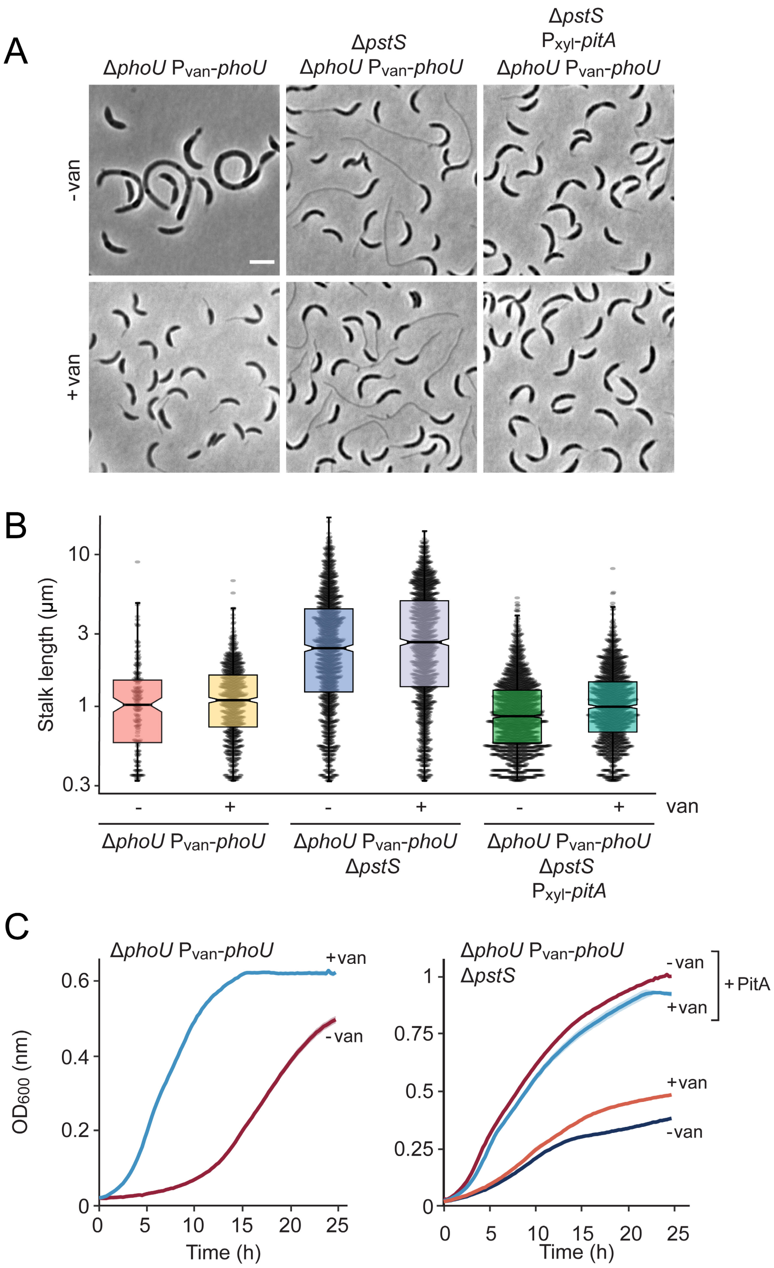

Supplementary Figure 12. PhoU is not a sensor for cytoplasmic P_i in *C. crescentus*. (A) Phase contrast images of *C. crescentus* Δ*phoU* P*van-phoU* (MAB310), Δ*pstS* Δ*phoU* P*van-phoU* (MAB539) and Δ*pstS* Δ*phoU* P*van-phoU* P*xyl-pitA* (MAB540) cells grown to exponential phase in PYE medium (+ P_i) in the presence or absence of vanillate. For the Δ*pstS* Δ*phoU* P*van-phoU* P*xyl-pitA* (MAB540) strain, xylose was added to the medium. Scale bar: 3 µm. **(B)** Combined beeswarm and box plots representing the distribution of stalk lengths in cultures of the strains shown in panel A. The boxes give the interquartile range, the notches indicate the median values, and the whiskers extend to the 5th and 95th percentile. Number of cells measured: MAB310 (-van): 190, MAB310 (+van): 887, MAB539 (-van): 1474, MAB539 (+van): 1590, MAB540 (-van, +xyl): 2019, MAB540 (+van, +xyl): 1613. **(C)** Growth behavior of the *C. crescentus* strains described in the legend to panel A with or without PhoU depletion in PYE medium. Lines show the average of two independent experiments. The edges of the shades indicate the values obtained in the two measurements.

SUPPLEMENTARY TABLES

Supplementary Table 1. *C. crescentus* and *E. coli* strains used in this study.

Strain	Genotype	Construction	Reference
C. crescentus			
CB15N	wild-type strain	aka NA1000	[1]
JK2	CB15N Δ*phoB*	In-frame deletion of CCNA_0296 (*phoB*) in CB15N using pJK3	This study
JK158	CB15N Δ*pstS*	Replacement of CCNA_01583 (*pstS*) with a Spec/Str^R marker in CB15N using pJK25	This study
MAB213	CB15N P_{xyl}::P_{xyl}-*pitA*	Integration of pMAB55 in CB15N	This study
MAB215	JK158 P_{xyl}::P_{xyl}-*pitA*	Integration of pMAB55 in JK158	This study
MAB221	MAB213 P_{pstS}-*lacZ*	Transformation of MAB213 with pMAB34	This study
MAB225	CB15N P_{CCNA_01606}-*lacZ*	Transformation of CB15N with pJR16	This study
MAB226	MAB213 P_{CCNA_01606}-*lacZ*	Transformation of MAB213 with pJR16	This study
MAB229	MAB215 P_{CCNA_01606}-*lacZ*	Transformation of MAB215 with pJR16	This study
MAB230	JK158 P_{CCNA_01606}-*lacZ*	Transformation of JK158 with pJR16	This study
MAB240	CB15N P_{pstS}-*lacZ*	Transformation of CB15N with pMAB34	This study
MAB241	MAB215 P_{pstS}-*lacZ*	Transformation of MAB215 with pMAB34	This study
MAB243	JK158 P_{pstS}-*lacZ*	Transformation of JK158 with pMAB34	This study
MAB257	CB15N P_{xyl}::P_{xyl}-*pitA*	Integration of pMAB74 in CB15N	This study
MAB258	JK2 P_{xyl}::P_{xyl}-*pitA*	Integration of pMAB74 in JK2	This study
MAB259	JK158 P_{xyl}::P_{xyl}-*pitA*	Integration of pMAB74 in JK158	This study
MAB309	MAB310 P_{xyl}::P_{xyl}-*pitA*	Integration of pMAB74 in MAB310	This study
MAB310	CB15N Δ*phoU* P_{van}-*phoU*	Integration of pMAB96 in CB15N and subsequent in-frame deletion of CCNA_0295 (*phoU*) using pMAB35	This study
MAB335	MAB213 P_{pstC}-*lacZ*	Transformation of MAB213 with pMAB104	This study
MAB336	CB15N P_{pstC}-*lacZ*	Transformation of CB15N with pMAB104	This study
MAB337	MAB215 P_{pstC}-*lacZ*	Transformation of MAB215 with pMAB104	This study
MAB341	JK158 P_{pstC}-*lacZ*	Transformation of JK158 with pMAB104	This study
MAB346	CB15N P_{CCNA_00486}-lacZ	Transformation of CB15N with pMAB105	
MAB349	CB15N P_{ppk1}-*lacZ*	Transformation of CB15N with pMAB106	This study
MAB350	MAB213 P_{ppk1}-*lacZ*	Transformation of MAB213 with pMAB106	This study
MAB351	MAB215 P_{ppk1}-*lacZ*	Transformation of MAB215 with pMAB106	This study
MAB352	JK158 P_{ppk1}-*lacZ*	Transformation of JK158 with pMAB106	This study
MAB539	MAB310 Δ*pstS*	Transduction of MAB310 with ΦCR30 lysate of JK158	This study
MAB540	MAB309 Δ*pstS*	Transduction of MAB309 with ΦCR30 lysate of JK158	This study
MAB541	CB15N P_{pstC}-*lacZ*	Transformation of CB15N with pMAB218	This study
MAB542	CB15N P_{ldpA}-*lacZ*	Transformation of CB15N with pMAB219	This study
MAB545	CB15N P_{stpX}-*lacZ*	Transformation of CB15N with pMAB222	This study
MAB546	CB15N P_{CCNA_00594}-*lacZ*	Transformation of CB15N with pMAB223	This study
MAB549	CB15N P_{CCNA_01636}-*lacZ*	Transformation of CB15N with pMAB226	This study
E. coli			
TOP10	F⁻ *mcr*A Δ(*mrr-hsd*RMS-*mcr*BC) Φ80*lac*ZΔM15 Δ*lac*X74 *rec*A1 *ara*D139 Δ(*ara leu*) 7697 *gal*U *gal*K *rps*L (Str^R) *end*A1 *nup*G		Invitrogen

Supplementary Table 2. General plasmids used in this work.

Plasmid	Description	References
pHP45	Plasmid carrying an Ω fragment for in vitro insertional mutagenesis (interposon) with a spectinomycin/streptomycin resistance gene.	[2]
pNPTS138	*sacB*-containing suicide plasmid used for double homologous recombination, Kan^R	M.R. Alley, unpublished
pPR9TT	RK2-based replicating plasmid for the construction of lacZ fusions. Cm^R	[3]
PVCHYC-4	Integrating plasmid for the construction of C-terminal fusions to mCherry under the control of P_{van}, Gen^R	[4]
pXCHYC-2	Integrating plasmid for the construction of C-terminal fusions to mCherry under the control of P_{xyl}, Kan^R	[4]
pXGFPC-5	Integrating plasmid for the construction of C-terminal fusions to GFP under the control of P_{xyl}, Tet^R	[4]

Supplementary Table 3. **Plasmids constructed in this work.**

Plasmid	Description	Construction
pJK3	pNPTS138 derivative for in-frame deletion of *phoB*	a) amplification of the *phoB* flanking regions from CB15N chromosomal DNA using primers oJK9+oJK10 (upstream) and oJK11+oJK12 (downstream)<br>b) restriction of the upstream fragment with HindIII and BamHI, restriction of the downstream fragment with BamHI and EcoRI<br>c) triple ligation with pNPTS138 cut with HindIII and EcoRI
pJK19	pNPTS138 derivative for in-frame deletion of *pstS*	a) amplification of the *pstS* flanking regions from CB15N chromosomal DNA using primers oJK26+oJK27 (upstream) and oJK28+oJK29 (downstream)<br>b) restriction of the upstream fragment with HindIII and BamHI, restriction of the downstream fragment with BamHI and EcoRI<br>c) triple ligation with pNPTS138 cut with HindIII and EcoRI
pJK25	pNPTS138 derivative for the replacement of *pstS* with an Ω cassette	a) restriction of pHP45 with BamHI and purification of the Ω cassette (Spec/Str^R) fragment<br>b) restriction of the pJK19 with BamHI<br>c) ligation of pJK19 with the Ω cassette fragment
pJR16	pPR9TT derivative carrying a translational fusion of the P_{CCNA_01606} with *lacZ*	a) amplification of the P_{CCNA_01606} from CB15N chromosomal DNA using primers oDK202 and oDK203<br>b) restriction of the fragment with KpnI and HindIII<br>c) ligation with pPR9TT cut with KpnI and HindIII
pMAB34	pPR9TT derivative carrying a translational fusion of the P_{pstS} with *lacZ*	a) amplification of the P_{pstS} from CB15N chromosomal DNA using primers oMAB81 and oMAB115<br>b) restriction of the fragment with BglII and HindIII<br>c) ligation with pPR9TT cut with BglII and HindIII
pMAB35	pNPTS138 derivative for in-frame deletion of *phoU*	a) amplification of the *phoU* flanking regions from CB15N chromosomal DNA using primers oMAB101+oMAB102 (upstream) and oMAB103+oMAB104 (downstream)<br>b) Overlap extension PCR and restriction with HindIII and EcoRI<br>c) ligation with pNPTS138 cut with HindIII and EcoRI
pMAB55	pXCHYC-2 derivative carrying *pitA*	a) amplification of *pitA* from TOP10 chromosomal DNA using primers oMAB180+oMAB181<br>b) restriction of the product with EcoRI and NheI<br>c) ligation into EcoRI/NheI-treated pXCHYC-2
pMAB74	pXGFP-5 derivative including *pitA*	a) amplification of *pitA* from TOP10 chromosomal DNA using primers oMAB272+oMAB181<br>b) restriction of the product with BglII and NheI<br>c) ligation into BglII/NheI-treated pXGFP-5
pMAB96	pVCFP-4 derivative including *phoU*	a) amplification of *phoU* from CB15N chromosomal DNA using primers oMAB105+oMAB106<br>b) restriction of the product with NdeI and NheI<br>c) ligation into NdeI/NheI-treated pVCFP-4
pMAB104	pPR9TT derivative carrying a translational fusion of the P_{pstC} with *lacZ*	a) amplification of the P_{pstC} from CB15N chromosomal DNA using primers oMAB331 and oMAB332<br>b) Gibson assembly with pPR9TT cut with KpnI and HindIII
pMAB105	pPR9TT derivative carrying a translational fusion of P_{CCNA_00486} with *lacZ*	a) amplification of P_{CCNA_00486} from CB15N chromosomal DNA using primers oMAB329 and oMAB330<br>b) Gibson assembly with pPR9TT cut with KpnI and HindIII
pMAB106	pPR9TT derivative carrying a translational fusion of P_{ppk1} with *lacZ*	a) amplification of P_{ppk1} from CB15N chromosomal DNA using primers oMAB333 and oMAB334<br>b) Gibson assembly with pPR9TT cut with KpnI and HindIII
pMAB218	pPR9TT derivative carrying a translational fusion of P_{pstC} with *lacZ*	a) amplification of P_{pstC} from CB15N chromosomal DNA using primers oMAB331 and oMAB527<br>b) Gibson assembly with pPR9TT cut with KpnI and XmaI
pMAB219	pPR9TT derivative carrying a translational fusion of P_{ldpA} with *lacZ*	a) amplification of P_{ldpA} from CB15N chromosomal DNA using primers oMAB525 and oMAB526<br>b) Gibson assembly with pPR9TT cut with KpnI and XmaI
pMAB222	pPR9TT derivative carrying a translational fusion of P_{stpX} with *lacZ*	a) amplification of P_{stpX} from CB15N chromosomal DNA using primers oMAB532 and oMAB533<br>b) Gibson assembly with pPR9TT cut with KpnI and XmaI
pMAB223	pPR9TT derivative carrying a translational fusion of P_{CCNA_00594} with *lacZ*	a) amplification of P_{CCNA_00594} from CB15N chromosomal DNA using primers oMAB534 and oMAB535<br>b) Gibson assembly with pPR9TT cut with KpnI and XmaI
pMAB226	pPR9TT derivative carrying a translational fusion of P_{CCNA_01636} with *lacZ*	a) amplification of P_{CCNA_01636} from CB15N chromosomal DNA using primers oMAB540 and oMAB541<br>b) Gibson assembly with pPR9TT cut with KpnI and XmaI

Supplementary Table 4. Oligonucleotides used in this work.

ID	Oligonucleotide	Sequence (5' to 3') [1]	Restriction site
oJK9	LB PhoB for	ATAAAGCTTCGGCGACGAGCGCCTGGACACCCTG	HindIII
oJK10	LB PhoB rev	ATGGATCCGTCTTCGTCTTCGACCACCAAAACG	BamHI
oJK11	RB PhoB for	ATGGATCCTCGGCGGGCTACTCGCTGGACATGG	BamHI
oJK12	RB PhoB rev	ATGAATTCGCTGGAGGCCTTGGTCGCCAGCCTG	EcoRI
oJK26	pstS LB for	ATGAATTCGGGGTGACCGAGTTCAAAAAGCCCAAG	EcoRI
oJK27	pstS LB rev	ATATGGATCCAGCGACGGTGGCGACCGCGCC	BamHI
oJK28	pstS RB for	ATATGGATCCAACGCCCTGACGCCGATGCCG	BamHI
oJK29	pstS RB rev	ATAAAGCTTCCTCAGGTCGCCGACGACGCGG	HindIII
oDK202	Pcc1537-for	TATGGTACCCAGAAGCCGACCAGCGACGACACCAG	KpnI
oDK203	Pcc1537-rev	TTTAAGCTTGCGCGGGAGATGACAGCCAGGGTTTTC	HindIII
oMAB81	PstSprom-F	AATTAAGATCTCTGACCATGGCGCTGGGCACA	BglII
oMAB101	PhoUUp-F	AATTGAATTCCGTCGAGGGCTCGATCCTGATCG	EcoRI
oMAB102	PhoUUp-R	CGTCGAGCTTGGGACGTTGCGAGTCAGGTGGGCCAGCTCTTCGCC	-
oMAB103	PhoUDo-F	GGCGAAGAGCTGGCCCACCTGACTCGCAACGTCCCAAGCTCGACG	-
oMAB104	PhoUDo-R	AATTAAGCTTCTTGCGAAGACGGCCGATGTGAA	HindIII
oMAB105	PhoUF-NdeI	AATTCATATGACCGAGCATACCGTCAAATCCTACGG	NdeI
oMAB106	PhoUR-NheI	AATTGCTAGCTTATTGCGACAGCACGTCGAGCTTGG	NheI
oMAB115	PstSprom-R(10cod)-2	AATATAAGCTTACGGTGGCGACCGCGCCGATG	HindIII
oMAB180	PitAF(EcoRI)	ATATGAATTCATGCTACATTTGTTTGCTGGCCTGGATTT	EcoRI
oMAB181	PitAR (NheI)	ATATGCTAGCTTACAGGAACTGCAAGGAGAGCCAGTACA	NheI
oMAB272	PitAF(BglII)	ATATAGATCTATGCTACATTTGTTTGCTGGCCTGGATTT	BglII
oMAB329	PCCNA_000486	ACAAAAGCTGGGTACCGGGGGCGGGCGGCG	-
oMAB330	PCCNA_00486-R	ATTCGATATCAAGCTTAACAGCGTGGTGGTGCGGAA	-
oMAB331	PpstC-F	ACAAAAGCTGGGTACCGTCTGACCGAACGCTTCTATCGGG	-
oMAB332	PpstC-lacZ-R	GAATTCGATATCAAGCTTAGGACGATAAGCGAAAGCCAGG	-
oMAB333	Pppk1-F	ACAAAAGCTGGGTACCCGAGCCTGATCTCTATCCCTACC	-
oMAB334	Pppk1-lacZ	AATTCGATATCAAGCTTTTGGCGGCGATCGGCAGG	-
oMAB527	PstC-R(XmaI)	CGACGGGATCCCCCGGGAGGACGATAAGCGAAAGCCAGGT	-
oMAB525	PldpA-F(KpnI)	ACTAAAGGGAACAAAAGCTGGGTACCTACGGCCGCAAGTACATGGGCA	-
oMAB526	PldpA-R(XmaI)	ACGACGGGATCCCCCGGGTTGAAGCGTTTGATCGCCATCGCC	-
oMAB532	pstpX-F(kpnI)	CACTAAAGGGAACAAAAGCTGGGTACCGACAAGGCCCCCTACGCGC	-
oMAB533	PstpX-R(xmaI)	CGACGGGATCCCCCGGGCGTATATTACGTCCAAACATCGGGTCCCC	-
oMAB534	Pccna_00594-F(kpnI)	CACTAAAGGGAACAAAAGCTGGGTACCCAGCTGGTCCCTGCGGTGTT	-
oMAB535	Pccna_00594-R(XmaI)	CGACGGGATCCCCCGGGAAGCTGGACAGGACAGGCATCAG	-
oMAB540	P01636-F(KpnI)	CACTAAAGGGAACAAAAGCTGGGTACCCTGATCCGCGTGCTGGAAATCCC	-
oMAB541	P01636-R(XmaI)	CGACGGGATCCCCCGGGTAGCGATCGAGGACGGACATGGC	-

SUPPLEMENTARY REFERENCES

1. Evinger M & Agabian N (1977) Envelope associated nucleoid from *Caulobacter crescentus* stalked and swarmer cells. *J. Bacteriol.* **132**, 294-301.
2. Prentki P & Krisch HM (1984) *In vitro* insertional mutagenesis with a selectable DNA fragment. *Gene* **29**, 303-313.
3. Santos PM, Di Bartolo I, Blatny JM, Zennaro E & Valla S (2001) New broad-host-range promoter probe vectors based on the plasmid RK2 replicon. *FEMS Microbiol. Lett.* **195**, 91-96.
4. Thanbichler M, Iniesta AA & Shapiro L (2007) A comprehensive set of plasmids for vanillate - and xylose-inducible gene expression in *Caulobacter crescentus*. *Nucleic Acids Res.* **35**, e137.
