# Supplementary material for: The cytoplasmic phosphate level has a central regulatory role in the phosphate starvation response of Caulobacter crescentus
Source: Commun Biol. 2024 Jun 26;7:772. doi: 10.1038/s42003-024-06469-y (PMC11208175; doi:10.1038/s42003-024-06469-y)
Supplement: Supplementary file 3 — Description of Additional Supplementary Files [file 42003_2024_6469_MOESM3_ESM.pdf]

Description of Additional Supplementary Files

File name: Supplementary Data 1.
Description: Analysis of the RNAseq data. The spreadsheets provide the raw and normalized RNA-seq data, pairwise comparisons of the datasets, and the lists of genes regulated by PhoB or the cytoplasmic phosphate level, respectively.

File name: Supplementary Data 2.
Description: Comparison of the list of PhoB-dependent genes established in this study with the previously identified PhoB regulon. The spreadsheet highlights similarities and differences between the direct PhoB regulon determined by Lubin et al. (2016) and the list of genes shown to be regulated, directly or indirectly, by PhoB in this study.

File name: Supplementary Data 3
Description: Gene ontology enrichment analysis. The spreadsheets list the 50 top-scoring biological functions among three sets of genes regulated by the cytoplasmic phosphate level in a manner dependent or independent of PhoB. Shown are the total number of annotated genes in the C. crescentus genome belonging to each individual GO term (annotated), the number of regulated genes categorized into each GO term (observed), the expected number if there was no enrichment for a specific GO term (expected), and the p values determined with a Fisher test.

File name: Supplementary Data 4
Description: Source data. The spreadsheets provide the source data and uncropped images used to generate the indicated figures.
